# Intracellularly delivered nanobody targeting the nucleocapsid protein effectively inhibits porcine deltacoronavirus replication

**DOI:** 10.1186/s13567-026-01738-6

**Published:** 2026-04-07

**Authors:** Chengyao Hou, Liangkai Liu, Runmin Kang, Shujun Liu, Yue Sun, Qinyuan Chu, Changwei Lei, Hongning Wang, Xin Yang

**Affiliations:** 1 Key Laboratory of Bio-Resource and Eco-Environment of Ministry of Education, Animal Disease Prevention and Green Development Key Laboratory of Sichuan Province, College of Life Sciences, Sichuan University, Chengdu, Sichuan China; 2 Sichuan Animal Science Academy, Chengdu, 610000 China

**Keywords:** Porcine deltacoronavirus, nucleocapsid protein, therapeutic nanobody, TAT peptide

## Abstract

Porcine deltacoronavirus (PDCoV) is an emerging enteropathogenic coronavirus that causes acute diarrhea in piglets and poses a potential zoonotic risk. Effective antiviral agents against PDCoV are currently lacking. In this study, we identified a nanobody, designated 62Nb, that specifically targets the nucleocapsid (N) protein of PDCoV and potently inhibits viral replication. Stable expression of 62Nb in porcine ST cells significantly reduced N protein levels, viral RNA copies, and progeny virus titers without affecting cell viability, indicating a robust antiviral effect. Mechanistic analysis revealed that 62Nb interferes with post-entry intracellular stages of the viral life cycle rather than viral attachment or entry. To enable extracellular delivery, we further engineered a TAT-fused nanobody (62Nb-TAT) that efficiently penetrated IPEC-J2 cells and maintained strong binding affinity to the N protein. The intracellularly delivered 62Nb-TAT markedly suppressed PDCoV replication in a dose-dependent manner, with an EC_50_ of 6.712 μM and a selectivity index of 11.98, demonstrating potent antiviral efficacy and favorable safety. Together, our results demonstrate that N protein–specific nanobodies represent a promising antiviral strategy against PDCoV infection, and that cell-penetrating peptide–mediated nanobody delivery provides a feasible approach for intracellular targeting of viral replication machinery.

**Graphical Abstract:**

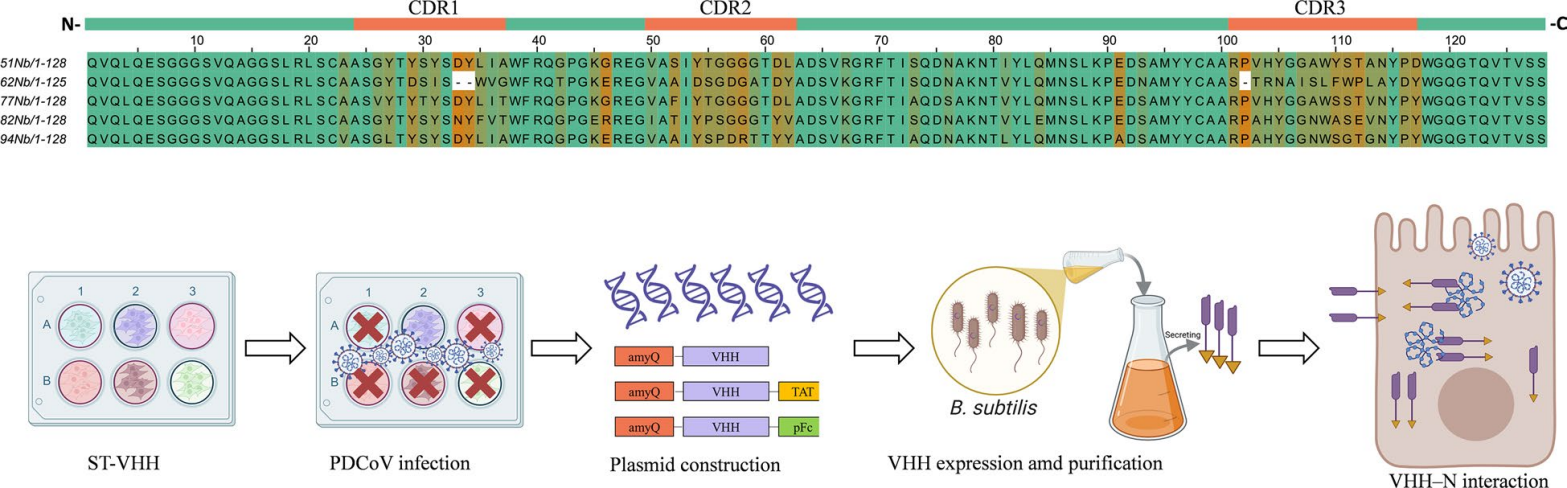

**Supplementary Information:**

The online version contains supplementary material available at 10.1186/s13567-026-01738-6.

## Introduction

Porcine deltacoronavirus (PDCoV) is an emerging enteropathogenic coronavirus belonging to the genus *Deltacoronavirus* [1, 2]. It causes acute diarrhea, vomiting, and dehydration in piglets, often leading to high mortality rates and substantial economic losses to the swine industry worldwide [3–5]. Since its first identification in 2012, PDCoV has been reported in multiple countries across Asia, North America, and Europe [1, 3, 4, 6–9]. Of particular concern, PDCoV RNA was detected in human samples in 2021, providing molecular evidence for potential cross-species transmission and raising concerns about its possible zoonotic threat [8].

Current strategies for PDCoV control primarily rely on biosecurity management and vaccination; however, vaccine-induced protection remains limited. Upon infection, PDCoV rapidly replicates in intestinal epithelial cells, causing severe intestinal damage, while effective antiviral therapeutics are still unavailable. The nucleocapsid (N) protein plays a central role in viral replication, assembly, and modulation of host immune responses, making it a promising target for antiviral intervention [10–13]. Molecular tools that target the N protein may help elucidate the mechanisms of PDCoV replication and offer new avenues for the development of antiviral strategies.

Nanobodies, single-domain antibody fragments derived from camelids, possess unique advantages such as small molecular size, high thermal stability, and ease of genetic engineering [14–17]. Compared with conventional antibodies, nanobodies are capable of recognizing hidden or conformational epitopes and have shown great potential in antiviral research [18–20]. Several nanobodies have been reported to inhibit viral infections by blocking viral entry or interfering with replication processes, such as those developed against SARS-CoV-2, PRRSV, and influenza A virus (IAV) [21–25]. However, to date, the antiviral functions of nanobodies against PDCoV have not been reported.

To enable intracellular targeting of viral proteins, efficient delivery of biologics across the plasma membrane is required. Cell-penetrating peptides (CPPs) represent a well-established class of short peptides capable of translocating diverse cargos, including proteins, peptides, and nucleic acids, into living cells. Among them, the trans-activator of transcription (TAT) peptide derived from human immunodeficiency virus type 1 (HIV-1) is one of the most extensively characterized CPPs and has been widely used to facilitate cytoplasmic delivery of functional proteins and antibody fragments. TAT-mediated delivery occurs primarily through energy-dependent endocytic pathways and has been successfully applied in antiviral research to enable intracellular access to viral replication machinery. Although TAT-based delivery is generally regarded as a proof-of-concept strategy rather than a clinically optimized system, its robustness, simplicity, and broad cell permeability make it a valuable tool for evaluating the intracellular antiviral potential of biologics targeting viral proteins that are otherwise inaccessible to conventional antibodies.

In our previous work, we successfully screened several high-affinity nanobodies targeting the PDCoV N protein, which exhibited excellent binding performance in vitro. Building on these findings, we constructed a porcine-derived ST cell line stably expressing the nanobody and systematically evaluated its impact on PDCoV replication. The results demonstrated that intracellular expression of 62Nb markedly inhibited PDCoV replication. Moreover, fusion of a cell-penetrating peptide enabled 62Nb to efficiently enter cells and exhibit potent antiviral activity in vitro. Further mechanistic studies revealed that 62Nb exerts its antiviral effect mainly by targeting the PDCoV N protein and interfering with the viral replication stage.

Taken together, this study provides the first evidence that an N protein-specific nanobody possesses potent antiviral activity against PDCoV at the cellular level. Our findings propose a nanobody-based intracellular delivery strategy for antiviral intervention, offering new theoretical and technical insights into molecular therapeutics against PDCoV infection.

## Materials and methods

### Reagents and materials

In-Fusion Snap Assembly Master Mix and PrimeSTA Max DNA Polymerase were obtained from Takara Biomedical Technology (Beijing) Co., Ltd. Trizol reagent was purchased from Thermo Fisher Scientific Inc. Puromycin, Geneticin (G418), doxycycline, and polybrene were purchased from GlpBio Technology (Shanghai) Co., Ltd. All-In-One 5 × RT Master Mix was supplied by Applied Biological Materials In (abm).

### Cell lines, virus strains and plasmids

Human embryonic kidney cells HEK293T (ATCC CRL-11268), swine testis cells ST (ATCC CRL-1746), and porcine intestinal epithelial cells IPEC-J2 (DSMZ ACC 701) were obtained from the indicated repositories and confirmed to be free of mycoplasma contamination prior to use. All cell lines were maintained in Dulbecco’s Modified Eagle Medium (DMEM; Gibco, Cat. No. 11965092, Carlsbad, CA, USA) supplemented with 10% fetal bovine serum (FBS; Gibco, Cat. No. 10099141), and 1% antibiotic–antimycotic solution (Gibco, Cat. No. 15240062). Cells were cultured at 37 °C in a humidified incubator with 5% CO_2_. Unless otherwise specified, all reagents used in this study were of analytical grade.

The PDCoV strain SCCZ18 (GenBank accession number MT985156) was preserved at the Sichuan Provincial Key Laboratory of Animal Disease Prevention and Green Development (Chengdu, China). The recombinant PDCoV-GFP virus was constructed using PDCoV SCCZ18 as the parental strain. Plasmids psPAX2 and pMD2.G were stored in our laboratory. The lentiviral vector pInducer20-Ev was derived from pInducer20 by removing redundant NotI and PstI restriction sites and replacing the Gateway cassette with a multiple cloning site containing NotI and PstI sites, thereby enabling doxycycline-inducible expression of C-terminal HA-tag VHH fragments. The plasmids PHT-62Nb, PHT-62Nb-TAT, and PHT-62Nb-pFc were synthesized by Sangon Biotech (Shanghai, China).

### Construction of stable ST cell lines expressing nanobodies

To investigate the inhibitory effects of the selected nanobodies against the PDCoV N protein, five stable ST cell lines with doxycycline-inducible nanobody expression were generated. The coding sequences of the five nanobodies were PCR-amplified using primers VHH-TY-F/R (Additional file 2) and inserted into the pInducer20-Ev expression vector using the In-Fusion Snap Assembly Master Mix.

For lentivirus production, HEK293T cells were seeded into 10-cm dishes at 60–70% confluency and co-transfected with the transfer plasmid (pInducer20-nanobody), the packaging plasmid psPAX2, and the envelope plasmid pMD2.G at a ratio of 4:3:1 using Lipofectamine 3000. After transfection, cells were incubated at 37 °C with 5% CO_2_. At 60 h post-transfection, the viral supernatant was collected, centrifuged at 500 × *g* for 5 min, and passed through a 0.45-µm PVDF filter (Millipore) to remove cell debris.

For transduction, ST cells were seeded in 6-well plates at 40–50% confluency and incubated with the lentiviral supernatant supplemented with polybrene (final concentration 8 µg/mL). After 48 h, the medium was replaced with fresh DMEM containing Geneticin (G418) at 500 µg/mL to select stably transduced cells. Selection pressure was maintained for 10–12 days until discrete colonies appeared. Positive clones were isolated by limiting dilution, expanded individually, and screened for nanobody expression by western blotting using anti-HA antibodies. Verified stable cell lines were expanded and cryopreserved in liquid nitrogen for subsequent experiments.

### Cell viability assay

To assess cell viability and proliferation of the stable nanobody-expressing ST lines, the Cell Counting Kit-8 assay was performed. Recombinant ST or IPEC-J2 cells (5 × 10^3^ cells/well) were seeded in 96-well plates and incubated for 1–7 days. Then, 10 µL of CCK-8 reagent was added per well and incubated for 1 h at 37 °C with 5% CO_2_. Optical density (OD) was measured at 450 nm using a microplate reader (Multiskan, Thermo Fisher). Cell viability was calculated as:

$${\mathrm{Cell}}\,{\mathrm{viability}}\,\left( \% \right)\, = \,\left[ {{{\left( {{\mathrm{OD}}\_{\mathrm{exp}}\, - \,{\mathrm{OD}}\_{\mathrm{blank}}} \right)} \mathord{\left/ {\vphantom {{\left( {{\mathrm{OD}}\_{\mathrm{exp}}\, - \,{\mathrm{OD}}\_{\mathrm{blank}}} \right)} {\left( {{\mathrm{OD}}\_{\mathrm{ctrl}}\, - \,{\mathrm{OD}}\_{\mathrm{blank}}} \right)}}} \right. \kern-0pt} {\left( {{\mathrm{OD}}\_{\mathrm{ctrl}}\, - \,{\mathrm{OD}}\_{\mathrm{blank}}} \right)}}} \right]\, \times \,{1}00\% .$$

### Indirect immunofluorescence assay (IFA)

To visualize antibody internalization and intracellular viral levels, indirect immunofluorescence assays were performed. IPEC-J2 or ST cells were seeded into 24-well plates and cultured to 70–80% confluence. Cells were infected with PDCoV at an MOI of 1 or treated with 10 μM antibody. After 2 h of infection, the medium was replaced with fresh medium and incubated for an additional 24 h at 37 °C with 5% CO_2_. Cells were then fixed with 4% paraformaldehyde, permeabilized with 0.1% Triton X-100 for 10 min, and blocked with 5% bovine serum albumin (BSA) for 1 h. Primary antibodies (e.g., mouse anti-PDCoV N, homemade porcine nanobodies, or anti-His-tag) were incubated overnight at 4 °C. After three PBS washes, Alexa Fluor 488/555-conjugated secondary antibodies (goat anti-mouse or FITC-conjugated goat anti-pig IgG) were applied for 1 h at room temperature in the dark. Nuclei were counterstained with DAPI, and images were captured under a fluorescence microscope (Leica, Germany).

### Western blot analysis

Western blotting was used to confirm antibody internalization and viral protein expression. Treated IPEC-J2 or ST cells were lysed in RIPA buffer containing protease and phosphatase inhibitors. Lysates were centrifuged at 12 000 × *g* for 15–20 min at 4 °C. Protein concentrations were determined using the BCA assay. Equal amounts of protein (20 μg per lane) were separated by 12% SDS-PAGE and transferred onto PVDF membranes (0.45 μm). After blocking with 5% non-fat milk in TBST for 1 h, membranes were incubated overnight at 4 °C with primary antibodies (anti-His, anti-PDCoV N, or nanobody). After washing, membranes were incubated with HRP-conjugated secondary antibodies for 1 h at room temperature. Signals were visualized using enhanced chemiluminescence (ECL) reagents and imaged with a ChemiDoc imaging system.

### Quantitative reverse transcription PCR (RT-qPCR)

Total RNA was extracted from PDCoV-infected cells at the indicated time points using TRIzol reagent. For each sample, cells were lysed directly in the culture plate with 1 mL TRIzol per well (6-well plate), mixed thoroughly, incubated for 5 min at room temperature, and combined with 200 µL chloroform. After vigorous shaking for 15 s, the mixture was incubated for 3 min and centrifuged at 12 000 × *g* for 15 min at 4 °C. The aqueous phase was transferred to a new tube, mixed with 500 µL isopropanol, incubated for 10 min, and centrifuged at 12 000 × *g* for 10 min. The RNA pellet was washed with 75% ethanol, air-dried, and dissolved in RNase-free water. RNA concentration and purity were assessed using a NanoDrop spectrophotometer.

For cDNA synthesis, 1 µg of total RNA from each sample was reverse-transcribed in a 20 µL reaction using the PrimeScript^™^ RT Reagent Kit following the recommended conditions: 37 °C for 15 min and 85 °C for 5 s.

Quantitative PCR was performed using TB Green^™^ Premix Ex Taq^™^ II in a 20 µL reaction containing 10 µL 2 × TB Green mix, 1 µL cDNA template, 0.4 µL each primer (10 µM), and nuclease-free water to volume. Amplification was carried out on a Bio-Rad CFX96 real-time PCR system with the following thermal cycling program:

95 °C for 30 s, followed by 40 cycles of 95 °C for 5 s and 60 °C for 30 s.

Melting curve analysis (65–95 °C) was included to confirm amplification specificity. PDCoV N gene-specific primers qPDN-F/R (Additional file 2) were used in all assays. Absolute viral RNA copy numbers were calculated based on a standard curve generated using the linear regression equation y = −3.3904x + 44.294.

All RT-qPCR data were obtained from at least three independent biological replicates and are presented as mean ± SD.

### Viral titration

To determine viral titers, infected cells were subjected to three freeze–thaw cycles, and the supernatants containing intracellular PDCoV were collected. Tenfold serial dilutions were prepared and inoculated onto confluent ST or IPEC-J2 cells (1 × 10^4^ cells/well) in 96-well plates, with eight replicates per dilution. After incubation at 37 °C for 5–7 days, cytopathic effects (CPEs) were observed daily. TCID_50_ values were calculated using the Kärber method and expressed as TCID_50_/mL.

### Flow cytometry

To evaluate the inhibitory effect of nanobodies on PDCoV replication, ST cells induced with doxycycline (1 μg/mL) were infected with PDCoV-GFP at various MOIs (10–320). Non-induced and mock-infected cells served as controls. After 24 h, cells were collected, washed, and analyzed by flow cytometry (BD Biosciences, USA) to determine the percentage of GFP-positive cells. Each condition was tested in triplicate.

### Determination of the affected stage of viral replication

To determine which stage of the PDCoV replication cycle was affected by nanobody expression, ST monoclonal cells stably expressing 62Nb were induced with DOX (1 μg/mL) and infected with PDCoV at an MOI of 1. Attachment: Cells were incubated with virus at 4 °C for 1 h, washed with PBS, and lysed for viral quantification. Entry: Cells were incubated at 37 °C for 1 h before lysis. Replication: Following 1 h adsorption at 37 °C, cells were washed and incubated in 2% DMEM containing trypsin for 8 h before harvest. Assembly/Release: Supernatants and cell lysates were collected 24 h post-infection to determine intracellular and extracellular viral loads.

### Pull-down assay

To verify the binding of nanobodies to the PDCoV N protein, 2 µg of 62Nb-Fc was incubated with Protein A/G magnetic beads for 1 h to allow coupling. Lysates from ST cells infected with PDCoV (1 MOI, 24 h) were incubated with the coupled beads at 4 °C for 2–4 h with rotation. Beads were washed three times with cold PBS containing 0.1% NP-40 and eluted by boiling in 2 × SDS loading buffer. Eluates were analyzed by western blot to detect the presence of N protein. Input lysates served as positive controls, and TGEV-N-pFc was used as a negative control antibody.

### Nanobody expression in *Bacillus subtilis*

Nanobody expression was conducted in *Bacillus subtilis* WB800N using the pHT43 plasmid (PHT-62Nb, PHT-62Nb-TAT, or PHT-62Nb-pFc), which carries a groE promoter and IPTG-inducible lac operator. The vector also includes an amyQ signal peptide for secretory expression. Positive clones were cultured in 200 mL of LB medium at 37 °C with shaking (250 rpm) until OD₆₀₀ = 0.6–0.8, induced with 1 mM IPTG, and incubated at 28 °C for 18 h. Supernatants were harvested by centrifugation, and proteins were purified using Ni–NTA affinity chromatography. Both Fc- and TAT-fused 62Nb variants were expressed in *Bacillus subtilis* for distinct experimental purposes, including biochemical characterization and evaluation of cellular delivery feasibility.

### Statistical analysis

Data were analyzed using GraphPad Prism 9. Statistical significance was determined by one-way analysis of variance (ANOVA), with *p* < 0.05 considered statistically significant. All experiments were performed in at least three biological replicates.

## Results

### Intracellular expression of 62Nb markedly suppresses PDCoV replication in ST cells

**Figure 1:**
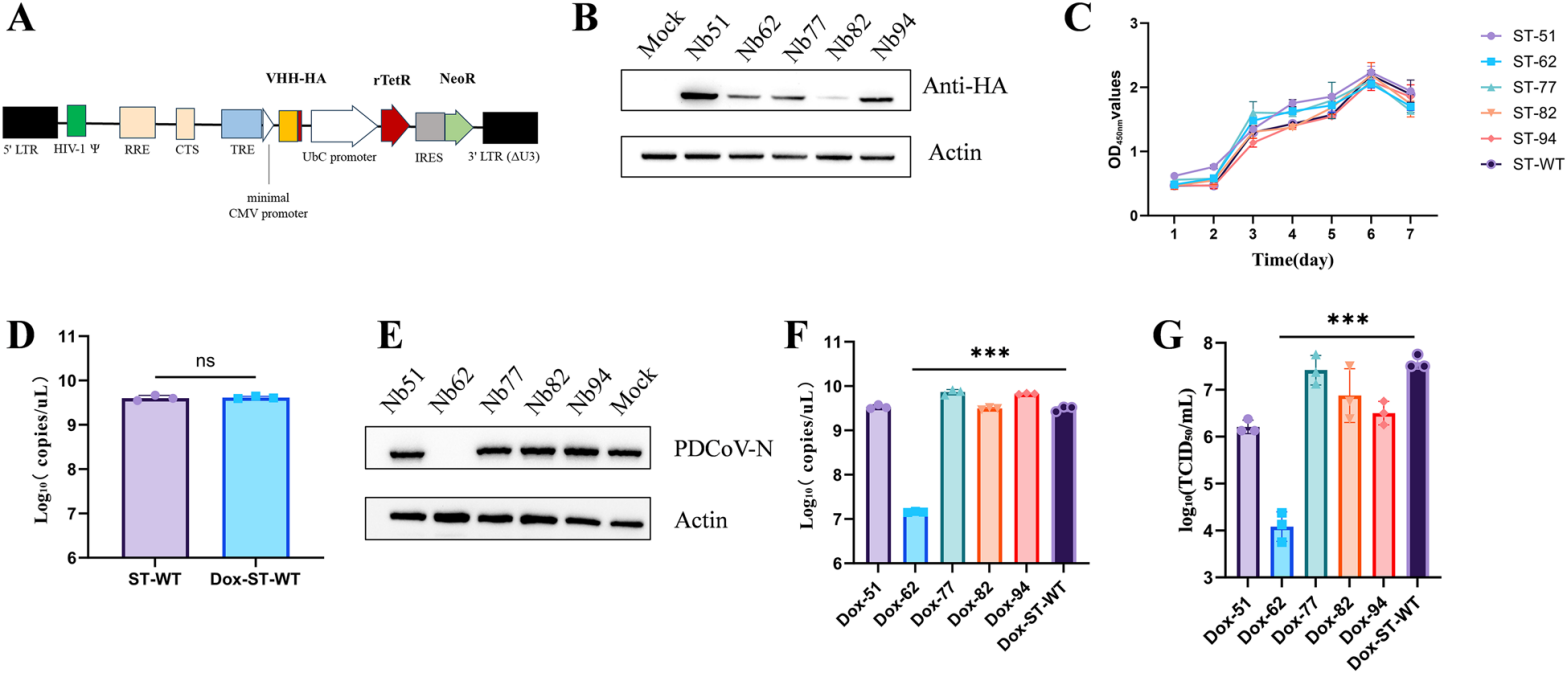
**Construction of ST cell lines stably expressing nanobodies (Nb) and their antiviral activity against PDCoV. A** Schematic representation of the inducible expression vector pInducer20-VHH-HA carrying HA-tagged nanobody. **B** Western blot analysis of inducible nanobody expression in five ST cell lines stably transduced by lentivirus. Nb (~15 kDa) was detected after 24 h induction with 1 µg/mL DOX, whereas no corresponding band was observed in wild-type ST cells. **C** Proliferation curves of the stable ST cell lines. **D** Effect of doxycycline (DOX) treatment on PDCoV replication in wild-type ST cells. ST-WT cells were treated with DOX under the same conditions used for inducible nanobody expression and subsequently infected with PDCoV. Viral RNA levels were quantified by RT-qPCR and are presented as log_10_ copies/µL. **E** Western blot detection of PDCoV N protein expression in cells infected with 1 MOI PDCoV for 24 h. **F** RT-qPCR analysis of viral mRNA levels in ST cells infected with 1 MOI PDCoV for 24 h. **G** Measurement of progeny virus titers (TCID_50_) in supernatants collected from ST cells 24 h post-infection and after three freeze–thaw cycles following 1 MOI PDCoV infection.

As shown in Figure 1A, an inducible lentiviral vector pInducer20-VHH-HA was successfully constructed to express HA-tagged nanobodies. Five stable ST cell lines generated via lentiviral transduction efficiently expressed the ~15 kDa nanobody upon induction with 1 µg/mL DOX, whereas no corresponding band was detected in wild-type ST cells (Figure 1B). Importantly, nanobody induction did not affect cell proliferation or morphology (Figure 1C).

To exclude the possibility that DOX itself influences PDCoV replication, wild-type ST cells were treated with DOX under the same conditions and subsequently infected with PDCoV. RT-qPCR analysis revealed no significant difference in viral RNA levels between DOX-treated and untreated ST cells (Figure 1D), indicating that DOX alone does not affect PDCoV replication.

When stable cell lines were infected with PDCoV at an MOI of 1, only 62Nb-ST cells exhibited no apparent cytopathic effect (CPE) at 24 h post-infection. Consistently, western blot and RT-qPCR analyses demonstrated a marked reduction in PDCoV N protein expression and viral RNA levels in 62Nb-ST cells compared with control cell lines (Figures 1E and F). TCID₅₀ assays further confirmed that progeny viral titers in 62Nb-ST cells decreased by approximately 3 log₁₀ units relative to wild-type ST cells (Figure 1G). Collectively, stable expression of 62Nb significantly inhibited PDCoV replication without affecting host cell growth, demonstrating potent intracellular antiviral activity.

### 62Nb exhibits potent antiviral activity against PDCoV

**Figure 2:**
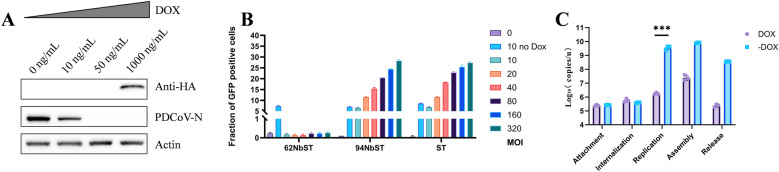
**Potent antiviral activity of 62Nb. A** Western blot analysis of DOX-inducible 62Nb expression and corresponding antiviral effects under a broad range of doxycycline (DOX) concentrations, used to evaluate dose-dependent nanobody induction and antiviral activity. **B** Flow cytometry analysis of viral inhibition in high-MOI (10–320) PDCoV-GFP infections. **C** RT-qPCR analysis of 62Nb effect on different stages of the PDCoV replication cycle.

To further evaluate the antiviral potential of 62Nb, we analyzed its expression and antiviral efficacy under various DOX concentrations. As DOX concentration increased, 62Nb expression rose accordingly, accompanied by progressively enhanced inhibition of PDCoV replication (Figure 2A). Notably, even under 50 ng/mL induction-where 62Nb expression was below the detection limit of western blot-PDCoV replication was still strongly suppressed, indicating exceptionally high antiviral potency.

To validate these findings, 62Nb-ST cells were infected with high-MOI (10–320) PDCoV-GFP and analyzed by flow cytometry. Without DOX induction, 62Nb-ST cells exhibited significant CPE with 7.23% GFP-positive cells. In contrast, upon 1 µg/mL DOX induction, the GFP-positive cell ratio remained below 0.3% even at an MOI of 320, comparable to the uninfected control (Figure 2B and Additional file 1). Conversely, 94Nb-ST and wild-type ST cells showed extensive CPE and high GFP-positive rates under the same conditions, confirming the strong inhibitory effect of 62Nb on PDCoV replication.

The use of extremely high MOI conditions in this experiment was intended to model severe viral challenge and to rigorously evaluate the robustness of antiviral activity under maximal infection pressure. The near-complete suppression of PDCoV-GFP infection even at MOIs up to 320 indicates that 62Nb-mediated inhibition is not readily overcome by increased viral input, highlighting its exceptional antiviral potency and stability under high replication stress.

To further explore the stage(s) of the PDCoV life cycle affected by 62Nb, viral dynamics were analyzed using stage-oriented infection assays. 62Nb had minimal effects on viral adsorption and entry. In contrast, viral RNA levels and progeny virus production were markedly reduced at post-entry stages, indicating that 62Nb interferes with intracellular steps of the viral life cycle occurring after entry, including viral genome replication and subsequent processes associated with virus production (Figure 2C). Collectively, 62Nb demonstrated robust antiviral potency, effectively blocking PDCoV replication even at low expression levels and under high-MOI viral challenge, highlighting its promise as a potent antiviral candidate.

### Interaction between *Bacillus*-expressed 62Nb and the N protein of PDCoV-infected cells

**Figure 3:**
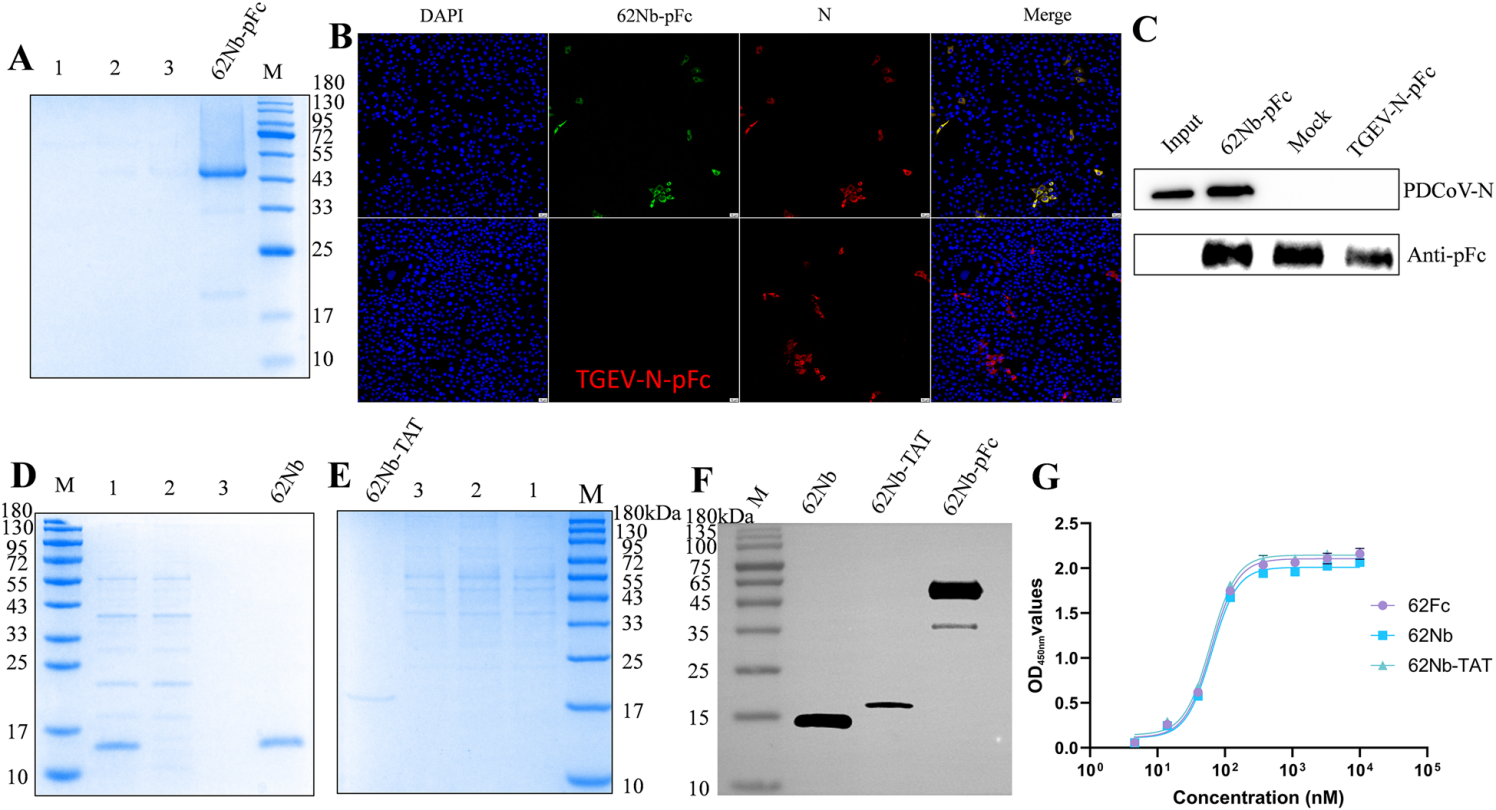
**Verification of 62Nb specificity and construction/characterization of fusion proteins. A** SDS-PAGE analysis of Fc-fused 62Nb (62Nb-pFc); lanes 1–3 represent contaminant wash, flow-through, and *Bacillus subtilis* secreted supernatant, respectively. **B** Immunofluorescence detection of 62Nb-pFc specificity for PDCoV N protein, with commercial mouse anti-N monoclonal antibody as positive control and TGEV-N-pFc as negative control. **C** Pull-down assay confirming specific interaction between 62Nb-pFc and PDCoV N protein; TGEV-N-pFc failed to pull down N protein. The pFc tag was directly recognized by commercially HRP-conjugated goat anti-pig secondary antibody. **D** SDS-PAGE analysis of *Bacillus subtilis* secreted unmodified 62Nb; lanes 1–3 correspond to secreted supernatant, flow-through, and wash fractions. **E** SDS-PAGE analysis of *Bacillus subtilis* secreted TAT-fused 62Nb (62Nb-TAT); lanes 1–3 correspond to secreted supernatant, flow-through, and wash fractions. **F** Western blot analysis of molecular weights: 62Nb-pFc (~46 kDa), 62Nb (~15 kDa), and 62Nb-TAT (~17 kDa), using mouse anti-His as primary antibody. **G** ELISA analysis of the binding activity of different nanobody forms against PDCoV N protein.

Based on the above findings, we hypothesized that 62Nb exerts its antiviral activity by recognizing and binding to the PDCoV N protein. To verify this, we constructed a pig Fc-fused nanobody (62Nb-pFc) and expressed it in *Bacillus subtilis*. SDS-PAGE and western blot analyses showed a molecular weight of ∼46 kDa (Figures 3A and F), and Fc fusion did not alter its affinity toward PDCoV N protein (Figure 3G). Immunodetection using 62Nb-pFc as the primary antibody specifically recognized PDCoV N protein, similar to the commercial mouse monoclonal antibody, whereas the TGEV-N-pFc control showed no reactivity (Figure 3B).

In pull-down assays, 62Nb-pFc successfully captured PDCoV N protein from infected cell lysates, while TGEV-N-pFc did not (Figure 3C), confirming a specific interaction between 62Nb-pFc and PDCoV N protein.

Given that the N protein is predominantly cytoplasmic, intracellular delivery is required for 62Nb to exert its antiviral function. To explore this, unmodified 62Nb and a TAT-fused version (62Nb-TAT) were expressed in *Bacillus subtilis*. SDS-PAGE and western blot showed molecular weights of ∼15 kDa and ∼17 kDa, respectively (Figures 3D–F). ELISA analysis revealed that both proteins retained strong binding activity toward PDCoV N protein (Figure 3G), indicating that TAT fusion did not compromise antigen recognition. These results provide a foundation for further in-cell and in-vivo functional evaluation.

### Efficient cellular uptake of 62Nb-TAT

**Figure 4:**
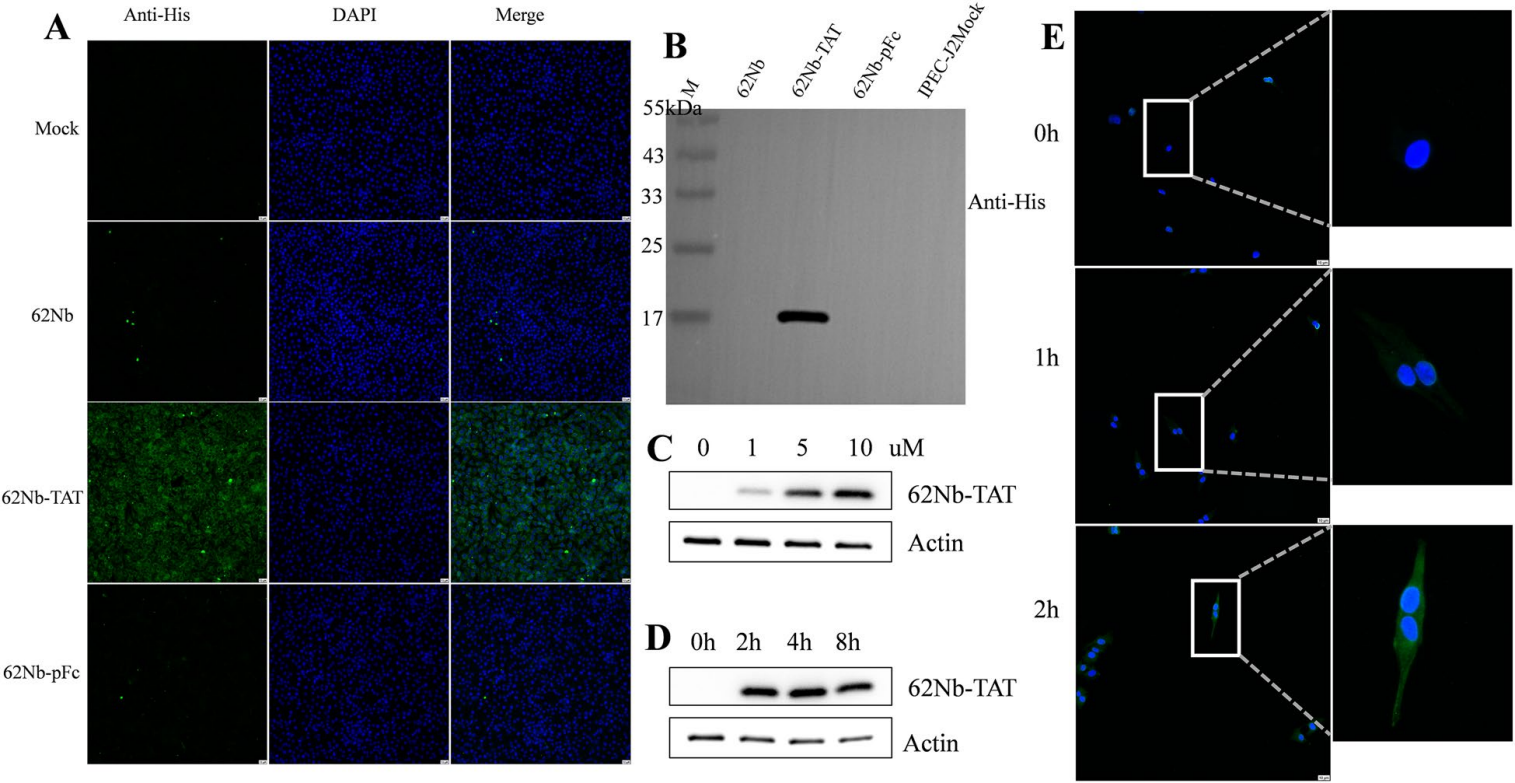
**Intracellular delivery of 62Nb fused with TAT peptide. A** Immunofluorescence analysis of intracellular distribution in IPEC-J2 cells treated with unmodified 62Nb, TAT-fused 62Nb (62Nb-TAT), or Fc-fused 62Nb (62Nb-pFc). **B** Western blot analysis of internalization of different nanobody forms in IPEC-J2 cells. **C** Western blot detection of intracellular 62Nb-TAT at various incubation concentrations (1–10 μM) to evaluate dose-dependency. **D** Western blot detection of intracellular 62Nb-TAT at different incubation times (2–8 h). **E** Time-course analysis of 62Nb-TAT internalization (0–2 h) and cytoplasmic localization using mouse anti-His as primary antibody.

To assess the cell-penetrating capability of the nanobodies, IPEC-J2 cells were incubated with 10 µM of 62Nb, 62Nb-TAT, or 62Nb-pFc for 12 h. Indirect immunofluorescence (IFA, Figure 4A) and western blot (Figure 4B) analyses revealed that only 62Nb-TAT efficiently entered cells and localized in the cytoplasm, whereas 62Nb and 62Nb-pFc showed no detectable intracellular signals.

Western blot quantification further demonstrated a dose-dependent increase in intracellular 62Nb-TAT levels (1–10 µM; Figure 4C). Time-course analysis showed that internalization reached a plateau within 2 h and remained stable thereafter (Figures 4D and E). These findings indicate that TAT fusion confers rapid and efficient cell penetration, allowing cytoplasmic localization required for antiviral action.

### 62Nb-TAT markedly inhibits PDCoV replication in IPEC-J2 cells

**Figure 5:**
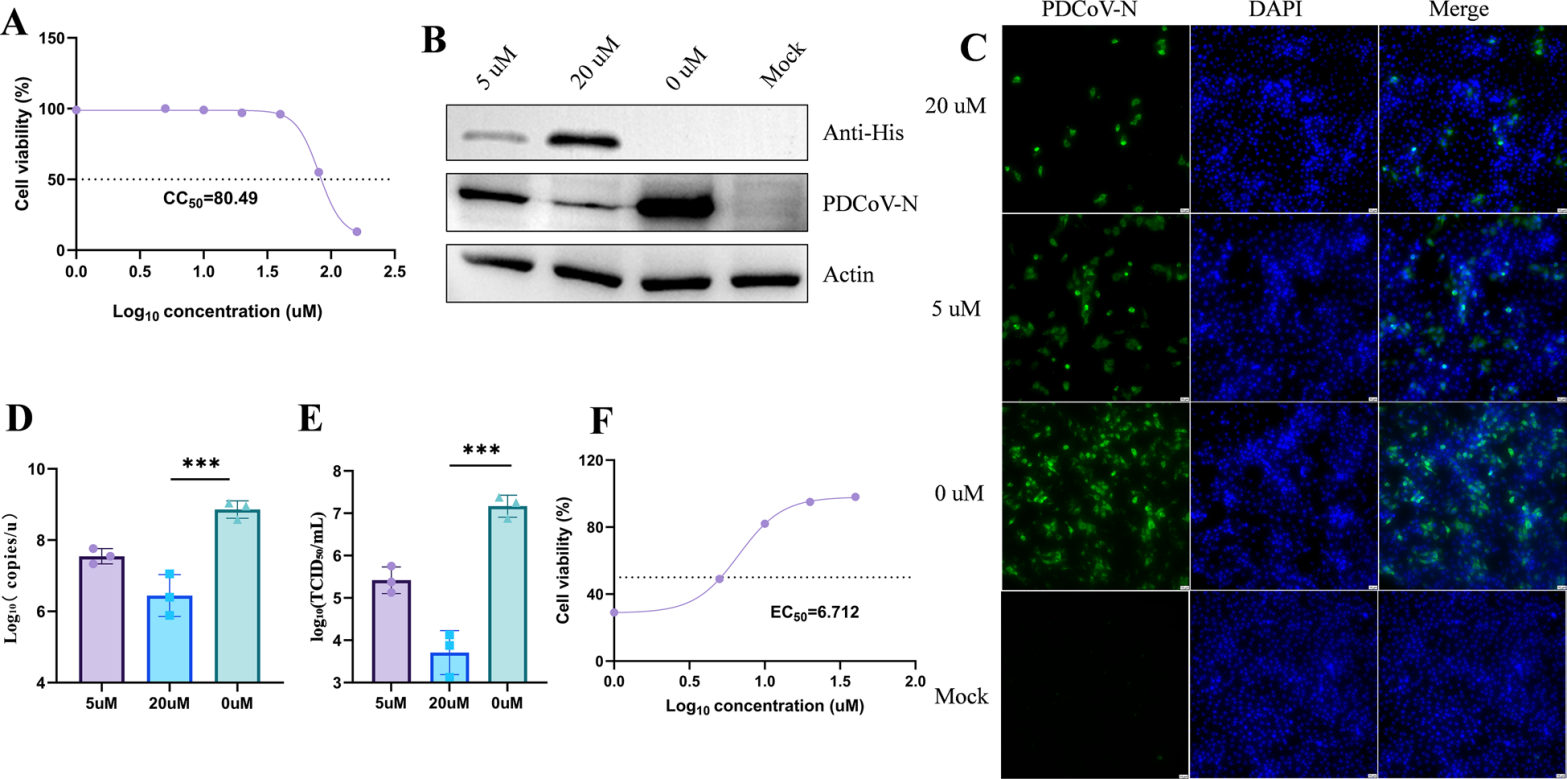
**In vitro antiviral activity of 62Nb-TAT against PDCoV. A** CCK-8 assay for half-maximal cytotoxic concentration (CC_50_) of 62Nb-TAT in IPEC-J2 cells. **B** Western blot analysis of PDCoV N protein expression in cells treated with 62Nb-TAT at 5 μM and 20 μM. **C** Immunofluorescence analysis of PDCoV-infected cells under different treatment concentrations. **D** RT-qPCR analysis of N gene mRNA levels. **E** TCID_50_ assay measuring progeny virus titers under different treatments. **F** CCK-8 assay determining half-maximal effective concentration (EC_50_) of 62Nb-TAT.

The cytotoxicity assay determined that the half-maximal cytotoxic concentration (CC_50_) of 62Nb-TAT was 80.49 µM, indicating good biocompatibility (Figure 5A). IPEC-J2 cells infected with PDCoV (MOI = 1) were treated with 5 µM or 20 µM 62Nb-TAT for 24 h. Western blot analysis showed dose-dependent inhibition of PDCoV N protein expression (Figure 5B), consistent with IFA observations showing a > 90% reduction in N-positive cells at 20 µM (Figure 5C). RT-qPCR revealed significant down-regulation of N gene transcripts (Figure 5D), and TCID₅₀ assays demonstrated a ~3-log₁₀ reduction in viral titers (Figure 5E).

The half-maximal effective concentration (EC_50_) of 62Nb-TAT was 6.712 µM (Figure 5F), yielding a selectivity index (SI = CC_50_/EC_50_) of 11.98. Together, these data indicate that 62Nb-TAT efficiently enters cells and strongly suppresses PDCoV replication in a dose-dependent manner with favorable safety and selectivity, providing compelling evidence of its therapeutic potential in vitro.

## Discussion

PDCoV is an emerging swine coronavirus that has attracted increasing attention due to its rapid transmission, frequently subclinical infections, and potential risk of cross-species transmission [4, 7–9]. Given the current lack of effective antiviral agents against PDCoV in swine production, intracellular nanobody-based strategies may represent a practical and promising alternative for disease control. Previous studies have demonstrated that coronavirus N protein plays a crucial role not only in viral assembly and RNA binding but also in modulating host cell signaling pathways, suppressing interferon responses, and regulating stress pathways, thereby promoting viral replication and immune evasion [11–13]. Compared with the frequently mutating spike (S) protein, the N protein is structurally more conserved with minimal cross-strain variation, making it an attractive target for antiviral intervention. In this study, we demonstrated that nanobodies targeting the N protein effectively inhibit PDCoV replication, further supporting the feasibility of N protein as a central target for antiviral design.

In our experiments, stable intracellular expression of 62Nb in porcine ST cells significantly reduced viral RNA levels and progeny virus titers. Importantly, potent antiviral activity was maintained even under high multiplicity of infection (MOI) conditions, reaching up to MOI 320, highlighting the robustness of its antiviral effect. From a biological perspective, the ability of 62Nb to retain antiviral efficacy under such extreme infection pressure suggests a substantial intracellular functional reserve, supporting its potential to withstand high viral burdens that may occur during acute infection or early outbreak stages. Notably, among the five nanobodies targeting the PDCoV N protein, only 62Nb exhibited pronounced antiviral activity in cell-based assays. A plausible explanation is that 62Nb recognizes a functionally critical epitope of the N protein directly involved in viral RNA binding, replication complex formation, or nucleocapsid assembly, whereas the other nanobodies may bind regions that are less essential for these processes.

Time-course analyses revealed that the antiviral activity of 62Nb primarily occurs at post-entry intracellular stages of the PDCoV life cycle, with minimal effects on viral adsorption and entry. It should be noted that, due to the intrinsic temporal overlap between viral genome replication and nucleocapsid assembly within infected cells, the experimental design employed in this study does not allow for strict separation of these two processes. Given the multifunctional roles of the coronavirus N protein in viral RNA synthesis, ribonucleoprotein complex formation, and virion assembly, 62Nb-mediated inhibition is therefore most likely to affect replication-associated steps and subsequent stages of virus production. Similar functional interference by nucleocapsid-targeting antibodies has been reported in other RNA viruses; for example, antibodies targeting the influenza A virus nucleoprotein inhibit viral replication by disrupting RNA binding and nucleocapsid assembly. Collectively, these observations suggest that targeting the N protein may represent a broadly applicable antiviral intervention strategy.

To further expand its therapeutic potential, we developed a TAT-fused form of 62Nb (62Nb-TAT) for extracellular delivery. This fusion protein efficiently entered the cytoplasm within a short time while maintaining high-affinity and specific binding to the N protein. Functional assays showed that 62Nb-TAT significantly suppressed PDCoV replication at low, non-toxic concentrations, reducing viral titers by over 3 log₁₀ units, with a selectivity index of 11.98, indicating an ideal therapeutic window. Compared with small-molecule inhibitors or interferon-based strategies, nanobodies offer superior specificity, stability, and low immunogenicity, making them suitable for targeting complex intracellular antigens. Nevertheless, it should be emphasized that TAT-mediated delivery in this study is primarily intended as a proof-of-concept approach to enable intracellular access of nanobodies targeting viral replication machinery. Although TAT fusion confers rapid and efficient cellular uptake in vitro, its non-specific internalization and limited tissue selectivity may pose challenges for precise in vivo applications. Accordingly, TAT is not presented as a clinically optimized delivery system, but rather as an experimental tool to validate the antiviral potential of intracellularly acting nanobodies. Future translational development may benefit from more targeted delivery strategies, such as biomaterial-assisted delivery or engineered probiotic systems, to enhance tissue specificity and in vivo stability while preserving antiviral efficacy. In this context, as demonstrated in the present study, the successful secretion of functional 62Nb and its derivatives by *Bacillus subtilis* supports the technical feasibility of this system as a production and exploratory delivery platform. At the current stage, this strategy is positioned as a proof-of-concept rather than a fully validated antiviral delivery system, with the primary aim of establishing a foundation for scalable nanobody production and exploring potential future development routes.

Another important implication of this study is the expansion of nanobody applications for intracellular antiviral research. Traditional antibodies are typically restricted to extracellular antigens and cannot target intracellular proteins critical for viral replication. By employing TAT-mediated delivery or intracellular expression, we overcame this limitation and enabled nanobodies to directly interfere with critical intracellular stages of the viral life cycle. Similar strategies have shown broad potential in other viral systems; for example, TAT-fused nanobodies effectively inhibited PRRSV Nsp9-mediated replication complex formation, and intracellular antibodies against IAV nucleoprotein blocked viral RNA synthesis [23, 25]. The PDCoV-N-62Nb model presented here not only enriches the paradigm of nanobody-mediated RNA virus intervention but also provides insights for the development of broadly applicable antiviral antibodies across coronaviruses.

Nevertheless, several aspects warrant further investigation. In the present study, the antiviral efficacy of 62Nb and 62Nb-TAT was primarily demonstrated in cell-based models, providing proof-of-concept evidence for intracellular targeting of PDCoV replication. Evaluation in piglets will therefore represent an important next step to determine in vivo pharmacokinetics, biodistribution, delivery feasibility, and protective efficacy in the context of PDCoV enteric infection. Such in vivo studies will also help define practical administration routes and dosing regimens suitable for field applications. In addition, structural characterization of the interaction interface between 62Nb and the PDCoV N protein may provide deeper mechanistic insights and facilitate rational optimization of nanobody design.

In summary, this study demonstrates that the N protein-targeting nanobody 62Nb possesses potent antiviral activity against PDCoV and can effectively suppress viral replication through intracellular expression or TAT-mediated delivery. These findings provide important experimental evidence for understanding PDCoV replication and for developing novel antiviral strategies. With continued advances in delivery technologies, N protein-targeting nanobodies may emerge as valuable molecular tools for combating emerging and re-emerging coronaviruses and for supporting integrated diagnostic and therapeutic approaches in animal coronaviruses.

## Supplementary Information

**Additional file 1. Flow cytometry analysis of viral inhibition in high-MOI (10-320) PDCoV-GFP infections.**

**Additional file 2. Primer sequences used in this study.**

## Data Availability

The datasets supporting the conclusions of this article are included within the article and its additional file.
