# Supplementary figures and images for: Intracellularly delivered nanobody targeting the nucleocapsid protein effectively inhibits porcine deltacoronavirus replication
Source: Vet Res. 2026 Apr 7;57:87. doi: 10.1186/s13567-026-01738-6 (PMC13214157; doi:10.1186/s13567-026-01738-6)

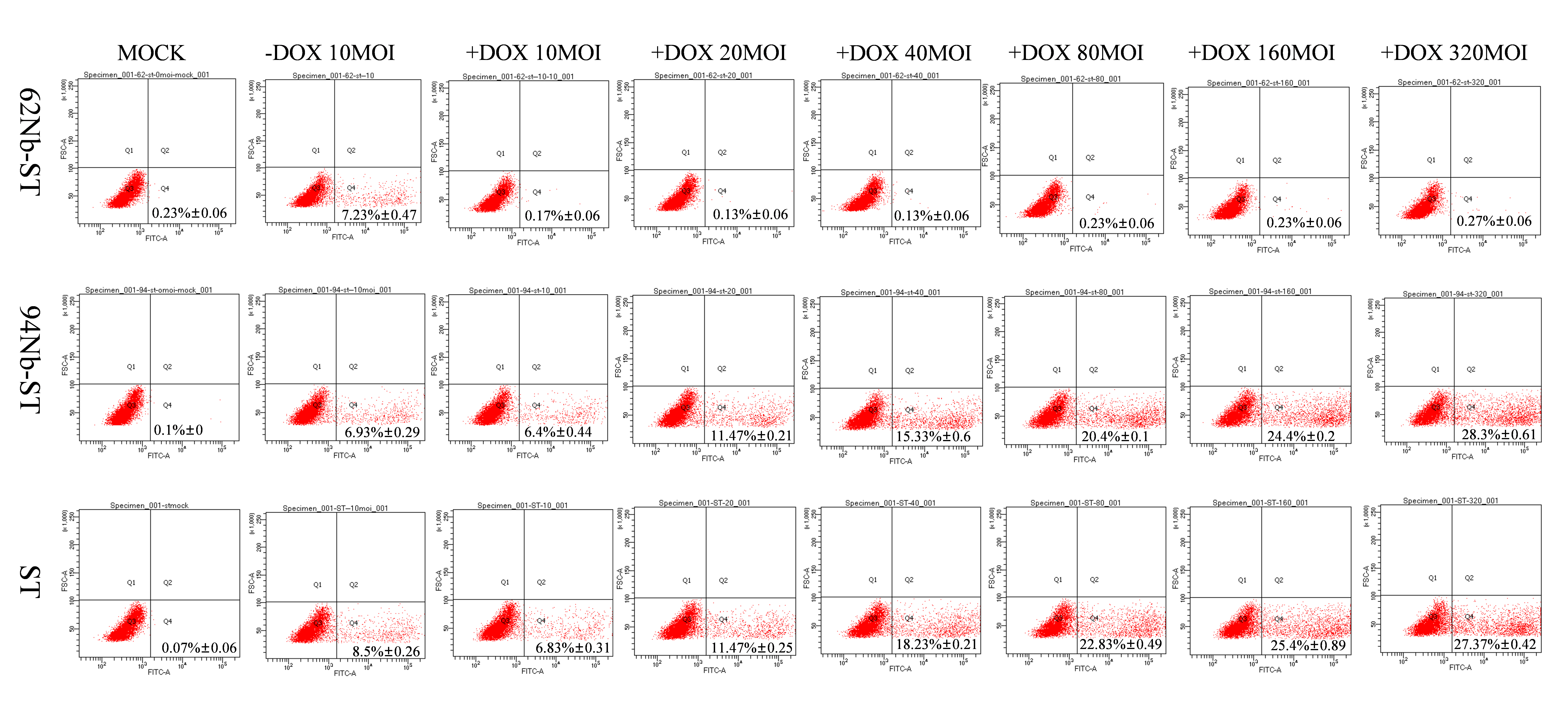

Supplement: Supplementary file 1 — Additional file 1. Flow cytometry analysis of viral inhibition in high-MOI (10-320) PDCoV-GFP infections. [file 13567_2026_1738_MOESM1_ESM.tif]
