# Supplementary material for: Intracellularly delivered nanobody targeting the nucleocapsid protein effectively inhibits porcine deltacoronavirus replication
Source: Vet Res. 2026 Apr 7;57:87. doi: 10.1186/s13567-026-01738-6 (PMC13214157; doi:10.1186/s13567-026-01738-6)
Supplement: Supplementary file 2 — Additional file 2. Primer sequences used in this study. [file 13567_2026_1738_MOESM2_ESM.docx]

**Additional file 2.** Primer sequences used in this study.

| Primer | Sequence(5′−3′) |
| --- | --- |
| VHH-TY-F | TCCAGTGTGGTGGAATTATGCAGGTGCAGCTGCAGGAGTC |
| VHH-TY-R | CGAACCGCGGGCCCTCTAGAGAGGAGACGGTGACCTGGGT |
| qPDN-F | GCTGCTACCTCTCCGATTCC |
| qPDN-R | GCTGATTGCCTGTGCCTCT |
